# Magnetosensitivity of Model Flavin–Tryptophan Radical Pairs in a Dynamic Protein Environment

**DOI:** 10.1021/acs.jpcb.5c01187

**Published:** 2025-06-04

**Authors:** Philip L. Benjamin, Luca Gerhards, Ilia A. Solov’yov, P. J. Hore

**Affiliations:** † Department of Chemistry, 6396 University of Oxford, Oxford OX1 3QZ, U.K.; ‡ Institute of Physics, 11233 Carl von Ossietzky Universität Oldenburg, Carl-von-Ossietzky Str. 9-11, 26129 Oldenburg, Germany; § Research Center for Neurosensory, Science, Carl von Ossietzky Universität Oldenburg, 26111 Oldenburg, Germany; ∥ Center for Nanoscale Dynamics (CENAD), Institute of Physics, Carl von Ossietzky Universität Oldenburg, Ammerländer Heerstr. 114-118, 26129 Oldenburg, Germany

## Abstract

Light-induced radical pairs in cryptochrome proteins located in the retina are thought to be the receptors at the heart of the magnetic compass sense of migratory songbirds. Reliable simulations of the performance of such sensors face several fundamental challenges. The quantum spin dynamics of large spin systems must be modeled for periods in excess of a microsecond including realistic local magnetic interactions that fluctuate on a picosecond to microsecond time scale as a result of thermal motion. Here we employ newly developed computational methods that combine explicitly time-dependent internal magnetic interactions, obtained from molecular dynamics simulations and electronic structure calculations, with efficiently and accurately modeled spin dynamics of multinuclear electron–nuclear spin systems. We identify the range of frequencies of molecular motions that are expected to have the greatest effects on the sensitivity of the proposed compass to the direction of an Earth-strength magnetic field and obtain new insights into the potential enhancements in detection sensitivity afforded by thermal modulations of electron–nuclear hyperfine interactions.

## Graphical abstract

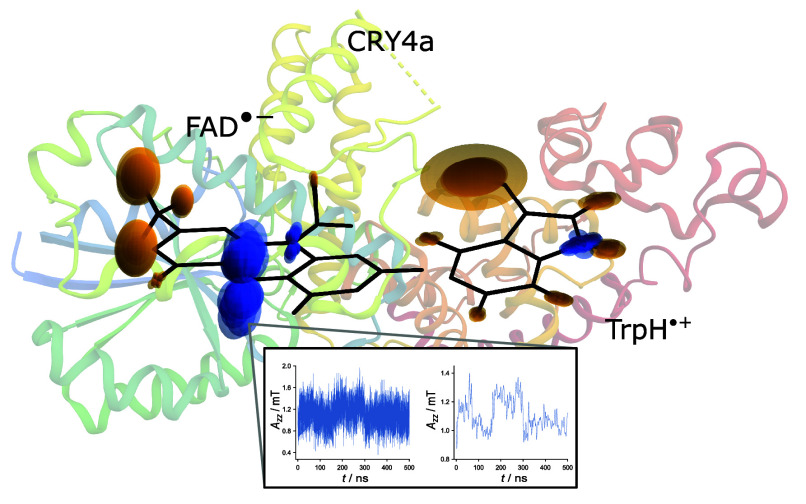

## 1 Introduction

Among other sources of directional information, migratory songbirds have a magnetic compass that guides their extraordinary, often intercontinental, seasonal journeys.^1–3^ The biophysical mechanism of this remarkable sense is thought to involve spin-correlated pairs of light-induced radicals formed from a flavin adenine dinucleotide (FAD) cofactor and a chain of four tryptophan (TrpH) residues in the protein cryptochrome-4a (CRY4a) located in the birds’ retinas.^4–8^ The sensitivity of this compass is determined by the magnetic interactions, electron spin relaxation and chemical reactivity of the radicals. Over the years, a number of attempts have been made to model the effects of the Earth’s magnetic field on these FAD^•–^-TrpH^•+^ radical pairs with the aim of learning more about the operation of the proposed sensor.^9–26^

Spin dynamics simulations of magnetic field effects on radical pairs in cryptochromes have generally considered the relevant spin interactions to be static, independent of time. The limitations of this approach have been exposed recently by studies in which molecular dynamics (MD) simulations were combined with electronic structure calculations to reveal the complex time-dependence of the magnetic interactions arising from thermal motion.^27,28^ The fluctuating local magnetic fields produced in this way relax the electron spins, tending to destroy the spin coherence with which the radical pairs are created.^29–31^ Despite intuitive reasoning that this would diminish the sensitivity to the direction of a weak external magnetic field, a few computational studies have reported increases in the available directional information as a result of the coupling between the spins and their fluctuating environment.^30,32,33^

Most descriptions of spin relaxation effects in the context of magnetoreception have been phenomenological, often restricted to “toy” spin systems with limited resemblance to radical pairs in cryptochrome. Expanding such calculations to more realistic systems presents a considerable computational challenge, not least because of the lengthy MD simulations and multiple electronic structure calculations required to obtain the time-dependence of the magnetic interactions. The hyperfine and dipolar couplings of the electron spins are modulated by protein motions at frequencies up to terahertz – around 10,000 times faster than the highest frequencies of coherent singlet–triplet interconversion. Explicit inclusion of such high-frequency modulations is essentially prohibitive using currently available spin dynamics simulation methods.

Here, we describe a new approach for including dynamic magnetic interactions in simulations of radical pairs in proteins. Using a range of spin systems, and exploring realistic regimes of molecular motions, we seek detailed insight into the effects of time-dependent interactions on the sensitivity of a radical pair magnetic compass. In doing so, we define a framework for including only the frequencies of molecular motions that have a significant effect on the quantum yields of the reaction products, an approach that enables large dynamic spin systems to be modeled with relative ease.

## 2 Methods

### 2.1 Spin Dynamics Simulations

The time-dependent spin interactions of radical pairs in a static external magnetic field **B** were modeled by means of the spin Hamiltonian:^19^

1 $$Ĥ\left(t\right)=\gamma_{e}B\cdot \left({Ŝ}_{1}+{Ŝ}_{2}\right)+{Ŝ}_{1}\cdot D\left(t\right)\cdot {Ŝ}_{2}+\underset{m}{\sum }{Î}_{1,m}\cdot A_{1,m}\left(t\right)\cdot {Ŝ}_{1}+\underset{m}{\sum }{Î}_{2,m}\cdot A_{2,m}\left(t\right)\cdot {Ŝ}_{2}$$

The first term is the Zeeman interaction of the electron spins with the magnetic field, with γ_e_ the electron gyromagnetic ratio. The second term is the electron–electron dipolar coupling specified by a time-dependent tensor, **D**(*t*). The final two terms are the electron–nuclear hyperfine couplings in the two radicals specified by tensors **A**_*i*,*m*_(*t*). The sums run over all nuclear spins in each radical. The electron exchange interactions of FAD-TrpH radical pairs in avian cryptochromes are considerably smaller than the dipolar couplings and are here assumed to be negligible.^8^

A radical pair containing *n* spin-1/2 and *m* spin-1 nuclei has a total of *Z* = 2^*n*^ × 3^*m*^ nuclear spin states. Whatever method is used, spin dynamics simulations inevitably become more demanding for larger *Z*. For small spin systems (e.g., *Z* ≤ 1000), we used a direct, time-propagation method,^34^ in which each nuclear spin state |ψ^(*j*)^⟩, *j* ∈ [1, *Z*], was propagated separately:

2 $$\left|\psi_{S}^{\left(j\right)}\left(t+\delta t\right)\right\rangle =e^{-iĤ\left(t\right)\delta t}\left|\psi_{S}^{\left(j\right)}\left(t\right)\right\rangle$$

where |ψ _S_^(*j*)^(0)⟩=|*S*⟩⊗|ψ ^(*j*)^⟩ for a radical pair initially in a singlet state, and *δt* is the time-step. The probability that a radical pair, formed in a singlet state at *t* = 0, is a singlet at a later time *t* is given by

3 $$P_{S}\left(t\right)=\frac{1}{Z}\overset{Z}{\underset{j=1}{\sum }}\left\langle \psi_{S}^{\left(j\right)}\left(t\right)\right|{\mathrm{P̂}}^{S}\left|\psi_{S}^{\left(j\right)}\left(t\right)\right\rangle$$

where *P̂* ^S^ is the singlet projection operator. Singlet and triplet pairs were assumed to recombine spin-selectively to form distinct singlet and triplet reaction products, with equal rate constants, *k*. The ultimate yield of the singlet product, Φ_S_, was obtained by integrating Equation 3 up to a time *T* at which the majority of the radicals had recombined:

4 $$\Phi_{S}=k\int_{0}^{T}e^{-kt}P_{S}\left(t\right)\;dt$$

The choice of *T* is a compromise. Smaller values may lead to inaccurate reaction yields while larger values mean longer simulation times. In all calculations here, the radical pairs had a lifetime of 1 μs (*k* = 10^6^ s^–1^), so that 0.7% and 0.009% of the radical pairs remain unreacted when *T* = 5 μs and *T* = 7 μs, respectively. Our experience has been that the correction method proposed and extensively tested in ref 28,

5 $$k\int_{0}^{\infty }e^{-kt}P_{S}\left(t\right)\;dt\approx \frac{k}{1-e^{-kT}}\int_{0}^{T}e^{-kt}P_{S}\left(t\right)\;dt$$

is satisfactory when *kT* ≥ 5. *T* was 7 μs for the spin system with 14 nuclei and 5 μs for all other simulations. The longer integration period in the former case was chosen to reflect the smaller values of ΔΦ_S_ expected for the larger spin system.

An Earth-strength magnetic field (∼50 μT) has a Larmor period of ∼700 ns which sets the time scale for geomagnetic compass sensing. If the lifetime of the radical pair (τ) is much less than 700 ns, there will be insufficient time for a 50 μT field to influence the spin dynamics and so alter the yields of the reaction products.^35^ We have therefore used a recombination rate constant *k* = 10^6^ s^–1^ so that τ = 1 μs.

The dependence of Φ_S_ on the orientation of the radical pair with respect to the magnetic field **B** was assumed to encode the directional information a bird would need to determine a compass bearing. Briefly, the magnetoreceptor molecules are thought to be immobilized and aligned in photoreceptor cells in the eyes so that birds would be able to derive a heading direction by comparing the magnetic signals from cells at different locations, and therefore with different orientations, in their retinas.^36–38^ This information is quantified by means of the singlet-yield anisotropy, ΔΦ_S_:

6 $$\Delta \Phi_{S}=\Phi_{S}{\left(\theta \right)}_{\max}-\Phi_{S}{\left(\theta \right)}_{\min}$$

where θ is the angle between **B** and the normal to the plane of the flavin group in the FAD-TrpH radical pair (Figure 1). Φ_S_(θ) was calculated for 0 ≤ θ ≤ π, keeping **B** perpendicular to the long axis of the flavin ring system.

**Figure 1:**
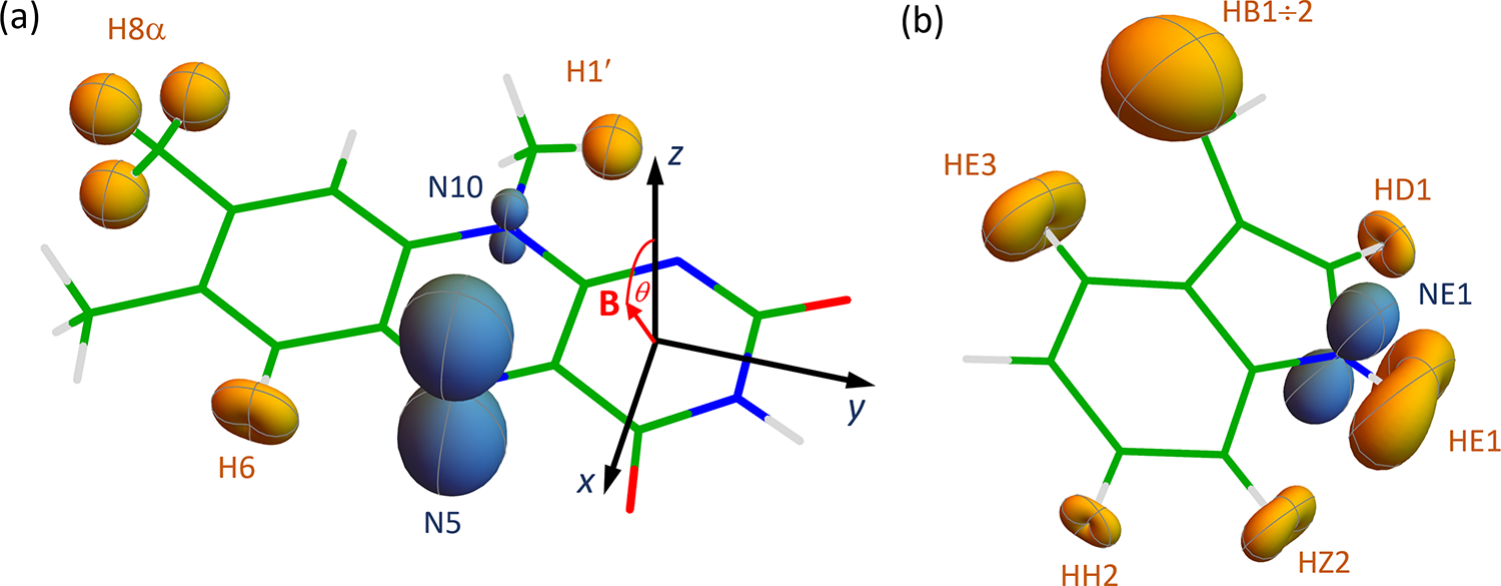
Representations of the ^1^H (yellow) and ^14^N (blue) hyperfine tensors in (a) the flavin part of the FAD^•–^ radical and (b) the indole part of the TrpH^•+^ radical. The external magnetic field was in the *xz*-plane of the flavin, making an angle θ with the *z*-axis. The hyperfine tensor of the HB1 proton in the TrpH^•+^ radical has been halved in (b). The relative orientation of the two radicals (not shown here) was taken from refs 28 and 42 .

For simulations of radical pairs containing 14 nuclear spins (*Z* = 55,296), we used the stochastic Schrödinger equation (SSE) method, recently implemented in the software package *MolSpin*, which allows the inclusion of arbitrary time-dependent spin interactions.^28,39,40^ This method has considerable advantages over perturbative approaches, such as Bloch–Redfield–Wangsness theory,^41^ which have previously been used to assess spin-relaxation effects in cryptochrome radical pairs.^30,31,40^

In the SSE method,^28,39^ the trace over the complete set of *Z* nuclear spin states in Equation 3 is replaced by a sum over *M* randomly sampled SU­(*Z*) coherent states, |Γ^(*j*)^⟩, so that the singlet fraction becomes

7 $$P_{S}\left(t\right)\approx \frac{1}{M}\overset{M}{\underset{j=1}{\sum }}\left\langle \Gamma_{S}^{\left(j\right)}\left(t\right)\right|{\mathrm{P̂}}^{S}\left|\Gamma_{S}^{\left(j\right)}\left(t\right)\right\rangle$$

with |Γ_S_^(*j*)^(0)⟩ defined and propagated in the same way as |ψ_S_^(*j*)^(0)⟩. This method of trace sampling allows *M* to be much smaller than *Z* due to the self-averaging nature of the SU­(*Z*) states, providing a massive computational advantage for large spin systems.^28,40^

Since both approaches require numerical propagation, the computation time scales inversely with the time-step, δ*t*, which must be less than half the period of the highest modulation frequency in *Ĥ*(*t*). The computational effort required to capture the highest frequency modulations arising from molecular motions therefore becomes prohibitive due to the enormous number of evaluations of Equations 3 or 7 that would be required. Including dynamic effects extracted from MD trajectories also becomes hugely expensive because of the need to calculate hyperfine tensors by density functional theory for a very large number of times, *t*.

### 2.2 Time-Dependence of Magnetic Interactions

In their computational study of the directional information available from model radical-pair compass sensors, Smith et al.^32^ sinusoidally modulated the distance between the radicals, causing the exchange interaction and the rate constant for singlet recombination to be time-dependent. The authors noted a strong dependence on the modulation frequency, with some frequencies resulting in substantial changes in the anisotropy of the product yields. Here, we investigate how ΔΦ_S_ is affected by three different kinds of fluctuations in the hyperfine and dipolar couplings: (a) artificial single-frequency modulation, (b) broadband-noise modulation, and (c) a more realistic time-dependence derived from an MD simulation.

For single-frequency modulation, a fraction Δ of each tensor element was modulated at frequency ν:

8 $$C_{ij}\left(t\right)=\left\langle C_{ij}\right\rangle \left[1+\Delta \sin\left(2\pi \nu t\right)\right]$$

where **C** represents either **A**_*i*,*m*_ or **D** and {*i*,*j*}∈{1,2,3}. All components of **A**_*i*,*m*_ and **D** were modulated with the same frequency, phase and amplitude factor, Δ.

Broadband noise modulation, comprised of *N* frequency components, each with a randomly sampled frequency, phase and amplitude, was implemented using

9 $$C_{ij}\left(t\right)=\left\langle C_{ij}\right\rangle \left[1+\frac{1}{\sqrt{N}}\overset{N}{\underset{n=1}{\sum }}\Delta_{n}\sin\left(2\pi \nu_{n}t+\alpha_{n}\right)\right]$$

The amplitude factors Δ_*n*_ were sampled from a normal distribution, $N\left(0,\sigma_{\Delta }\right)$ , with zero mean and standard deviation σ_Δ_, while the frequencies ν_*n*_ and phases α_*n*_ were sampled from uniform distributions, *U*(ν_1_,ν_2_) and *U*(0,2π), respectively. Each tensor element, *C*_*ij*_(*t*), was modulated independently except that *C*_*ij*_(*t*) = *C*_*ji*_(*t*) to preserve the hermiticity of *Ĥ*(*t*). For both Equations 8 and 9, the time-step, δ*t*, was 50 ps.

The static components of the hyperfine and dipolar tensors, ⟨**C**⟩, used for both single-frequency and broadband-noise modulation, are listed in Table S1 in the Supporting Information. For the more realistic simulations of radical pairs with 14 nuclear spins, the time-dependence of these interactions was obtained explicitly (rather than stochastically) using frames taken at intervals of δ*t* = 50 ps from a 953 ns all-atom MD simulation. The time-averaged components of the various tensors are listed in Table S2. The MD simulation, an extension of simulations reported in previous work,^8,27,28^ was based on a homology model of European robin CRY4a derived from the crystal structure of pigeon CRY4a (PDB ID: 6PU0),^43^ with system preparation carried out using the VIKING platform.^44^ Simulations were performed using NAMD^45,46^ with the CHARMM36 force field, TIP3P water model, and 50 mM NaCl in an isothermal environment maintained at 310 K using a Langevin thermostat.^47–51^ The 953 ns simulation used here started life as a 200 ns production simulation, preceded by 5 ns dynamic equilibration.^8^ Grüning et al.^27^ used this well equilibrated structure as the starting point for a 400 ns simulation, which was then extended to 953 ns by Pažėra et al.^28^

### 2.3 Removal of High-Frequency Noise Components

High-frequency components were removed from ***A***_*i*,*m*_(*t*) and ***D*** (*t*) using the following procedure, illustrated in Figure 2. Each *C*_*ij*_(*t*) time-series was Fourier transformed using *SciPy*’s implementation of the fast Fourier transform algorithm and the amplitudes of components with frequencies above a predetermined cutoff, ν_cutoff_, were set to zero. After inverse Fourier transformation, the time-step was changed to *δt* = 500 ps by retaining every 10th data point. In this way all hyperfine-induced S↔T interconversion frequencies and molecular motions at frequencies up to *min*(ν_cutoff_, 1 GHz) were reliably sampled.

**Figure 2:**
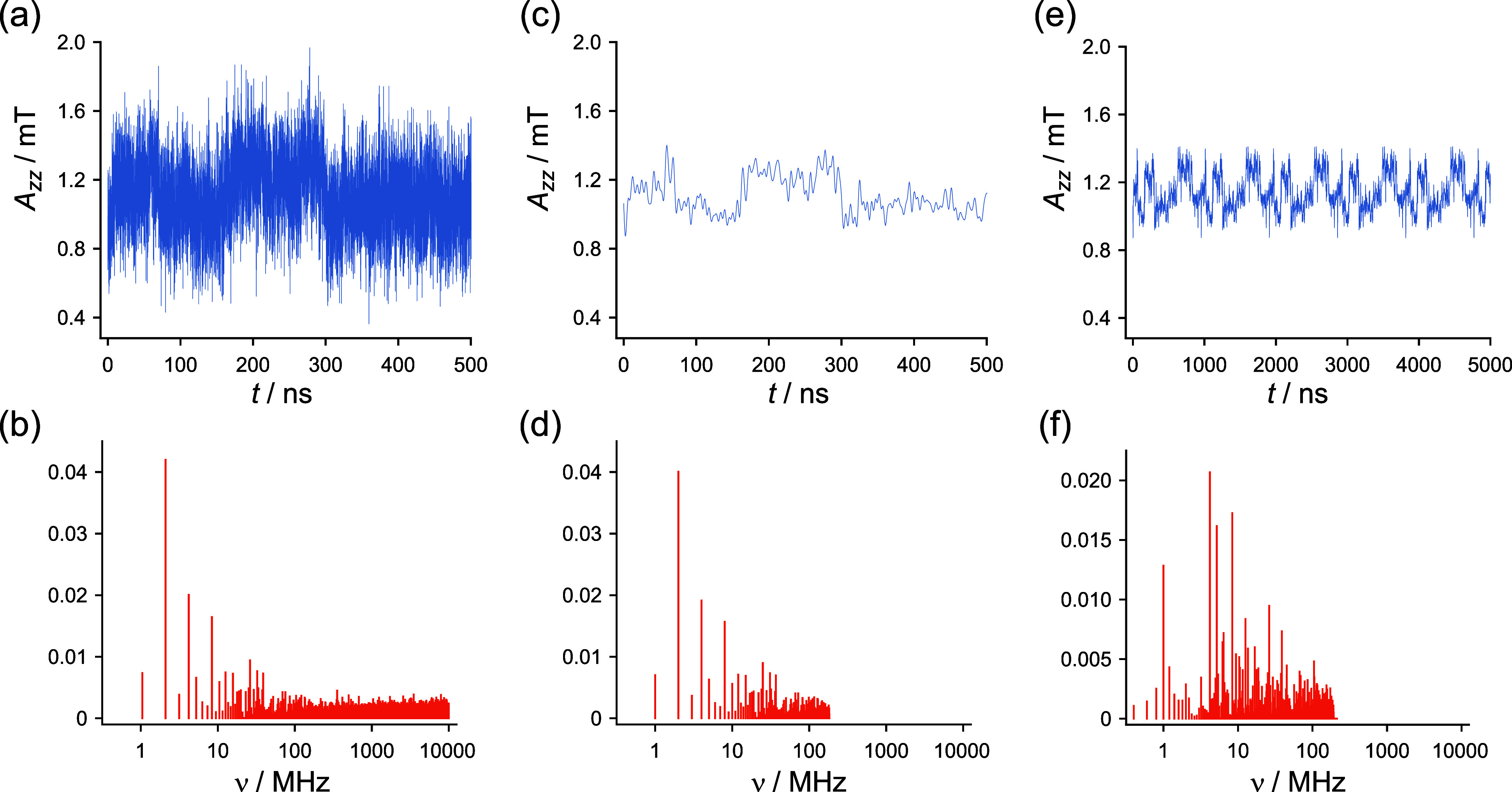
Illustrative MD-generated time-series and their Fourier transforms for the *A*_*zz*_ component of the hyperfine interaction of the N5 nitrogen in the FAD^•–^ radical. (a) First 500 ns of the time-series derived from the 953 ns MD trajectory of European robin CRY4a,^28,42^ with δ*t* = 50 ps. (b) Fourier transform of (a). (d) Data of (b) with frequency components above 200 MHz removed. (c) First 500 ns of the inverse Fourier transform of (d), with δ*t* = 500 ps. (e) Time-series extended to ∼5 μs by 6-fold concatenation of the 953 ns time-series followed by filtering to remove components above 200 MHz. (f) Fourier transform of (e). All 953 ns of (c) were used to extend the time-series to ∼5 μs (Figures 7 and 8) or ∼7 μs (Figure 9). No apodization or zero-padding were used in the production of this figure or in the removal of the high-frequency components. See Section S4 of the Supporting Information for further details.

The hyperfine and dipolar modulations obtained from the 953 ns MD simulation were extended to the end of the integration period *T* (Equation 4) by 8-fold, end-to-end concatenation of the *δt* = 50 ps traces, followed by the filtering process described above (Figure 2). This approach introduces artificial frequency components below ∼1 MHz, and may slightly distort the amplitudes at other frequencies. Nevertheless, we feel this is a pragmatic solution to modeling the dynamics of a large protein without requiring excessive computational resources. The alternative, a single MD simulation spanning the full 7 μs integration window of Equation 4, would have had considerable computational demands given that ∼150 ns per day is achievable on our current system. On top of this, the DFT calculations of the hyperfine tensors would have increased the computational cost to a level impractical for the scope of this work.

### 2.4 Calculation of Hyperfine and Dipolar Interactions from MD Simulations

The time-dependent magnetic interactions of the radicals were calculated using a 953 ns all-atom MD simulation.^28,42^ FAD^•–^ and TrpH^•+^ radical fragments were extracted from 19,060 MD frames at 50 ps intervals and dangling bonds were capped with geometry-optimized hydrogen atoms. The hyperfine interactions were obtained using the B3LYP functional and the EPR-II basis set implemented in Gaussian 16 (ref 52) as described in refs 28 and 42 . The dipolar interactions were calculated using the point-dipole approximation:

10 $$D_{jk}\left(t\right)=\frac{\mu_{0}\hbar {\gamma_{e}}^{2}}{4\pi }\left[\frac{\delta_{jk}}{r{\left(t\right)}^{3}}-\frac{3r_{j}\left(t\right)r_{k}\left(t\right)}{r{\left(t\right)}^{5}}\right]$$

in which *r*(*t*) = |**r**(*t*)| and **r**(*t*) = (*r*_*x*_(*t*), *r*_*y*_(*t*), *r*_*z*_(*t*)) is the vector connecting the centroids of the isoalloxazine flavin group of FAD^•–^ and the indole group of TrpH^•+^. μ_0_ is the vacuum permeability and δ_*jk*_ is the Kronecker delta.

## 3 Results

### 3.1 Artificial Noise: Frequency Dependence

We start with a simple toy model of the FAD-TrpH radical pair in cryptochrome to assess the kinds of changes that can be expected when the internal magnetic interactions are modulated at different frequencies. Each of the radicals contains a single spin-1 nucleus and the static anisotropic hyperfine interactions are those of the nitrogen nuclei at position N5 in the FAD^•–^ radical and position NE1 in the TrpH^•+^ radical (see Figure 1 for atom labels and Table S1 for hyperfine and dipolar tensors). The time-dependent hyperfine and dipolar interactions, generated using Equation 8 or 9, were input to *MolSpin* as “trajectory files”,^28,53^ to enable calculation of the singlet yield, Φ_S_, and its anisotropy, ΔΦ_S_, using Equations 2–6. Codes for generating such time-dependent magnetic interactions are available at the GitHub repository (https://github.com/Das0Mann/MolSpin) and will be included in the next release of *MolSpin.* The lifetime of the radical pair was 1 μs and the magnetic field was 50 μT. Two examples of *MolSpin* input files can be found in Section S3 of the Supporting Information.

Figure 3a shows the dependence of ΔΦ_S_ on the frequency of monochromatic modulation (Equation 8 with an amplitude factor, Δ = 0.1). Also shown (horizontal gray line) is the value of ΔΦ_S_ calculated using Δ = 0, i.e. for purely static interactions, and ν = ν_max_ ≈ 81 MHz (vertical red line), which is the difference between the highest and lowest eigenvalues of the static spin Hamiltonian. Modulating the hyperfine and dipolar interactions at frequencies below ν_max_, either increases or decreases ΔΦ_S_; modulations at frequencies above ν_max_ have no effect on ΔΦ_S_. The origin of this behavior appears to mirror the effects of external radiofrequency magnetic fields on the spin-state populations and spin-coherences of radical pairs.^54–56^ Frequencies above ν_max_ are ineffective at altering the singlet–triplet interconversion because they are “off-resonance”: they do not match the difference between any pair of spin eigenvalues and so cannot affect the spin dynamics or, therefore, the reaction yields. For both of the model spin systems studied here, ν_max_ is dominated by the hyperfine interactions. For example, for the toy spin system, changing the static dipolar parameter from −0.824 mT to −0.347 mT, corresponding to an increase in the radical–radical separation from 1.5 to 2.0 nm, reduces ν_max_ by ∼5%. Variation of the direction of the 50 μT magnetic field changes ν_max_ by ∼3%. Additional calculations of ν_max_ are given in Section S2 of the Supporting Information.

**Figure 3:**
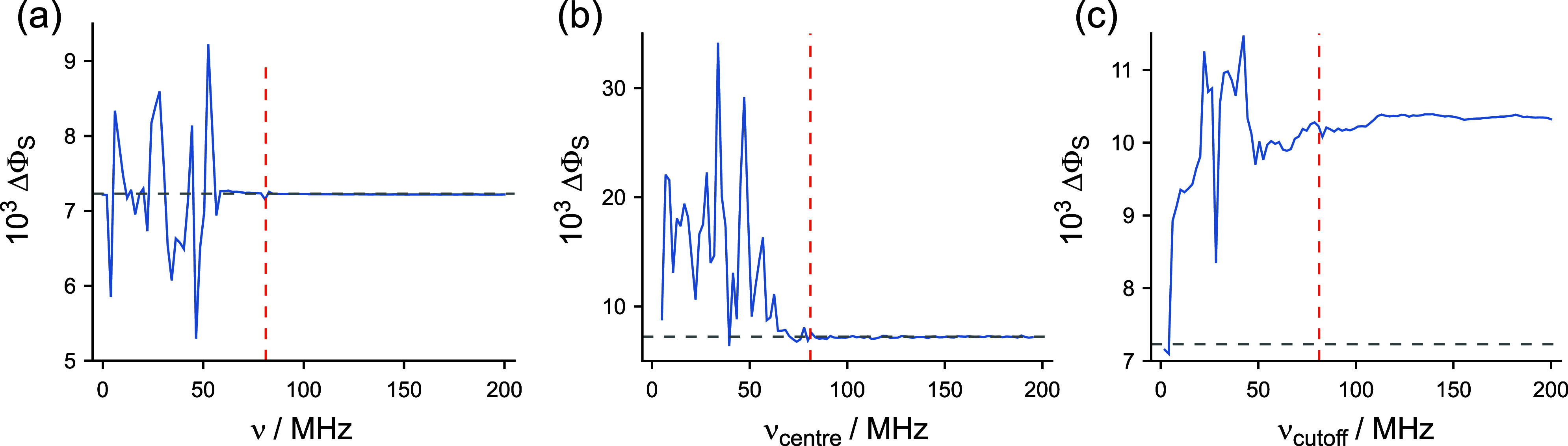
Changes in ΔΦ_S_ arising from modulation of hyperfine and dipolar interactions for a toy radical pair model. (a) Monochromatic modulation at frequency ν (Equation 8). (b) Broadband noise modulation at frequencies (ν_center_ – 5 MHz) ≤ ν ≤ (ν_center_ + 5 MHz) (Equation 9). (c) Broadband noise modulation at frequencies 0 ≤ ν ≤ ν_cutoff_ (Equation 9). The gray dashed lines give the value of ΔΦ_S_ for time-independent interactions. The red dashed lines show the value of ν_max_, the difference between the largest and the smallest eigenvalues of the static part of the spin Hamiltonian in Equation 1.

Figure 3b shows the frequency-dependence of ΔΦ_S_ for broadband-noise modulation (Equation 9 with σ_Δ_ = 0.1, *N* = 100, and 10 MHz-wide frequency bands centered at 5 ≤ ν_center_ ≤ 195 MHz. As found for single-frequency modulation (Figure 3a), frequencies below ν_max_ alter ΔΦ_S_ whereas those above ν_max_ do not.

For Figure 3c, Equation 9 (with σ_Δ_ = 0.1 and *N* = 1000) was used to generate broadband noise in the range 0–200 MHz which was then filtered, as described in Section 2.3, to remove components with frequencies above ν_cutoff_, with 0 ≤ ν_cutoff_ ≤ 200 MHz. In this case, ΔΦ_S_ reaches a plateau once ν_cutoff_ ≈ ν_max_; addition of higher-frequency components had little effect. In contrast to single-frequency modulation (Figure 3a), noise modulation of the internal magnetic interactions increases ΔΦ_S_ (by ∼50% in Figure 3c) compared to the purely static case.

In summary, all three calculations shown in Figure 3 suggest that only modulation frequencies below ν_max_ have a significant effect on the reaction yield, Φ_S_(θ) and its anisotropy, ΔΦ_S_.

The choice of 0.1 for the modulation amplitudes (Δ and σ_Δ_) in Figure 3 was somewhat arbitrary. To judge the generality of those results, we repeated these calculations using stronger and weaker modulations, and the same toy model, with one nitrogen hyperfine interaction in each radical. The simulations shown in Figure 3c were repeated using 10 values of σ_Δ_ between 0.01 and 1.0, and the same procedure to remove components above ν_cutoff_. These values of the modulation amplitude were chosen to range from unrealistically small (1%) to unrealistically large (100%). As can be seen from Figure 4, for all but the largest amplitudes (σ_Δ_ > ∼0.5), although ΔΦ_S_ was strongly affected by modulation frequencies up to ν_max_, it changed much less when frequencies beyond ν_max_ were included. This confirms that the span of the eigenvalue spectrum of the static spin Hamiltonian is indeed a reliable upper limit on the modulation frequencies expected to modify ΔΦ_S_. For all the radical pair models considered below, ν_max_ was smaller than 200 MHz: in subsequent simulations, we therefore sampled the frequencies in Equation 9 from a uniform distribution between 0 and ν_cutoff_ = 200 MHz.

**Figure 4:**
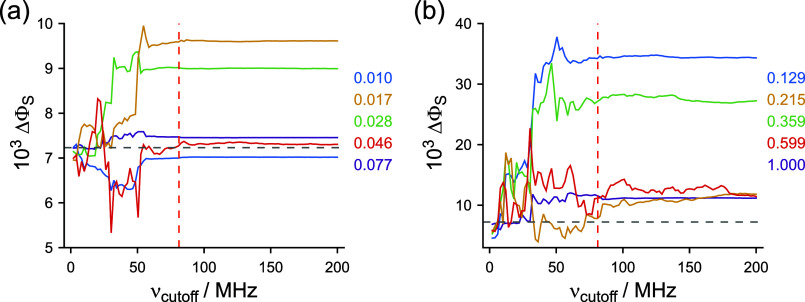
Changes in ΔΦ_S_ arising from broadband-noise modulation of hyperfine and dipolar interactions for a toy radical pair model. See text for details. The values of σ_Δ_ are as indicated. Results for (a) the five smaller values of σ_Δ_ and (b) the five larger ones. The gray and red dashed lines are as in Figure 3.

### 3.2 Artificial Noise: Amplitude-Dependence

The calculated values of ΔΦ_S_ in Figures 3b,c and 4 show a complex frequency-dependence for frequencies below ν_max_, partly because of the small number of eigenvalues of the static spin Hamiltonian (*Z* = 9) and partly because every calculation of ΔΦ_S_ had its own set of noise parameters (Δ_*n*_, ν_*n*_, δ_*n*_; *n* ∈ [1, *N*] in Equation 9). The variation in the directional signal as a result of thermal noise is relevant in practice because signals generated by a set of similarly oriented magnetoreceptor molecules at each location within the retina will almost certainly need to be averaged to improve signal-to-noise. This averaging could be spatial, temporal or both: over a set of identically oriented molecules in the same cell, sets of molecules in adjacent cells, and/or over periods of several minutes during which a number of molecules would be photoexcited at different times. Each radical pair would have its own random thermal fluctuations in hyperfine and dipolar interactions, and therefore different ΔΦ_S_.

To explore the effects of noise in more detail, we calculated the directional dependence of Φ_S_(θ) for the toy model, using σ_Δ_ = 0.108, ν_cutoff_ = 200 MHz and 16 different realizations of the noise. The results are shown in Figure 5a. Even for this relatively conservative choice of σ_Δ_, there is considerable variation in the range of Φ_S_(θ) values and the positions of the maxima and minima. Nevertheless, the average, ⟨Φ_S_(θ)⟩, of the 16 calculations shows a clear anisotropy that could be suitable for direction finding.

**Figure 5:**
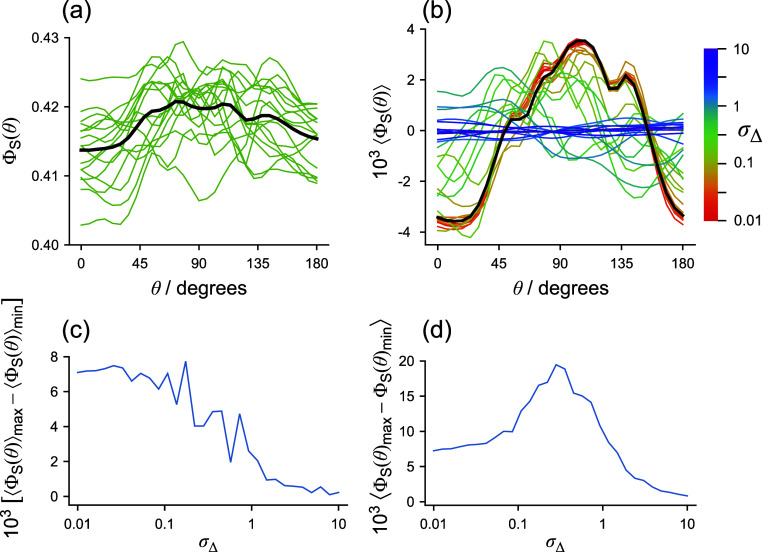
Effects of noise on radical pair reaction yields. See text for details. (a) 16 simulations of the singlet yield, Φ_S_(θ), as a function of magnetic field direction (green lines). A different realization of the noise was used for each of the 16 traces. The black line is the average singlet yield, ⟨Φ_S_(θ)⟩. (b) Dependence of the average singlet yield anisotropy, ⟨Φ_S_(θ)⟩, on magnetic field direction for values of the noise amplitude σ_Δ_ between 0.01 and 10. Each trace is the average of 16 calculations (as in (a)) with the isotropic component subtracted. (c, d) Averaged singlet yield anisotropies, as described in the text.

To see how this average signal varies with the modulation amplitude, Figure 5b shows the θ-dependence of ⟨Φ_S_(θ)⟩ for noise standard deviations σ_Δ_ between 0.01 and 10. For the smallest σ_Δ_, this average is, as expected, close to that calculated for time-independent magnetic interactions. As σ_Δ_ is increased, the fluctuating local magnetic fields cause the spins to relax such that ⟨Φ_S_(θ)⟩ flattens out and tends toward the value (0.25) appropriate for thermal equilibrium. This behavior is summarized in Figure 5c in which the anisotropy of the average signal, ${\left\langle \Phi_{S}\left(\theta \right)\right\rangle }_{\max}-{\left\langle \Phi_{S}\left(\theta \right)\right\rangle }_{\min}$ , is plotted against σ_Δ_. Values of σ_Δ_ greater than ∼0.1 produce a distinct reduction in the sensitivity of the radical pair as a compass sensor.

In agreement with Figures 3b,c and 4, almost all of the 16 individual traces in Figure 5b have a larger range of Φ_S_(θ) values than does the calculation with static magnetic interactions. Figure 5d shows the σ_Δ_-dependence of ⟨Φ_S_(θ)_max_ − Φ_S_(θ)_min_⟩, obtained by averaging the anisotropies of the 16 calculations for each value of σ_Δ_. Now, instead of a loss of detection sensitivity as the noise amplitude is increased, this average signal has a maximum at σ_Δ_≈0.3, about twice the size of the value for essentially static interactions (when σ_Δ_ = 0.01) suggesting that thermal motions could have the effect of boosting the sensitivity of the magnetic sensor.

A discussion of which average, ⟨Φ_S_(θ)⟩_max_ – ⟨Φ_S_(θ)⟩_min_ (Figure 5c) or ⟨Φ_S_(θ)_max_ − Φ_S_(θ)_min_⟩ (Figure 5d), is more appropriate in the context of magnetoreception is deferred to the Discussion section.

### 3.3 Molecular Dynamics Noise

The toy model shows how dynamic hyperfine and dipolar interactions can affect the directional information available from a radical pair sensor (Figures 3–5). However, those simulations have limited applicability not only because artificial noise was used to modulate the interactions but also because only 2 of the 27 nuclear spins in the FAD-TrpH radical pair were included.^22^ As the next step toward a more realistic description of a cryptochrome magnetoreceptor, we used the time-dependence of atomic positions extracted from an MD simulation of European robin (*Erithacus rubecula*, *Er*) CRY4a to calculate fluctuations in hyperfine and dipolar interactions using density functional theory for the former and Equation 10 for the latter.^28,42^ At the same time, we expanded the spin system of the radical pair to a maximum of 14 hyperfine interactions. The 953 ns, all-atom MD simulation of *Er*CRY4a was extended to 7 μs and frequency components above 200 MHz were removed using the procedures described in Section 2.3. The time-step for propagating |ψ_S_^(*j*)^(*t*)⟩ (Equation 2), δ*t* = 500 ps, is short enough to capture both the coherent singlet–triplet interconversion of the radical pairs and all relevant frequencies at which atomic motions modulate the hyperfine and dipolar interactions.

An analysis of the hyperfine tensors of the FAD^•–^ and TrpH^•+^ radicals is given in Figure 6. Once the frequency components above 200 MHz have been removed, the average fluctuation in these interactions is of the order of 10%. This can be compared with Figure 4 which shows that broadband-noise modulation at frequencies above ν_max_ has little effect on ΔΦ_S_ when the modulation amplitude is less than 10% (i.e., σ_Δ_ < 0.1). We interpret this as sufficient justification for excluding frequencies above 200 MHz from the hyperfine tensors calculated from MD trajectories.

**Figure 6:**
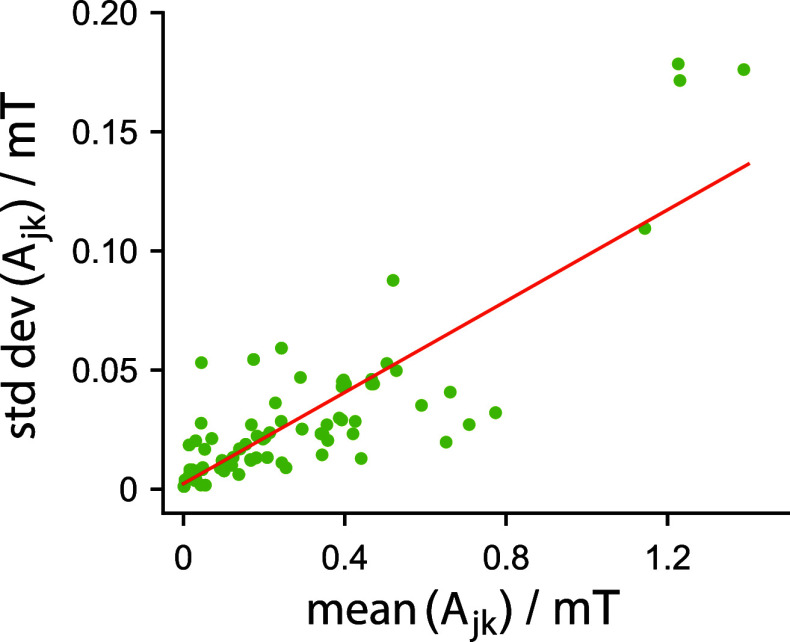
Standard deviations of FAD^•–^ and TrpH^•+^ hyperfine tensor components plotted against the magnitudes of the corresponding means. Data were calculated from the MD simulation with δ*t* = 500 ps after removal of frequencies above 200 MHz. The nuclei included in this plot are those listed in Table S2. The red line is a linear least-squares fit with gradient 0.096 and intercept 0.0024 mT.

For the 2-nitrogen toy model, Figure 7 shows the equivalent of Figure 3c, but now using MD-based modulations of hyperfine and dipolar interactions instead of artificial noise. Once again, we see that increasing the cutoff frequency beyond ν_max_ produces no further change in ΔΦ_S_. The enhancement in ΔΦ_S_ relative to the static case (a factor of ∼ 12) is considerably larger than in Figure 3c (∼1.5).

**Figure 7:**
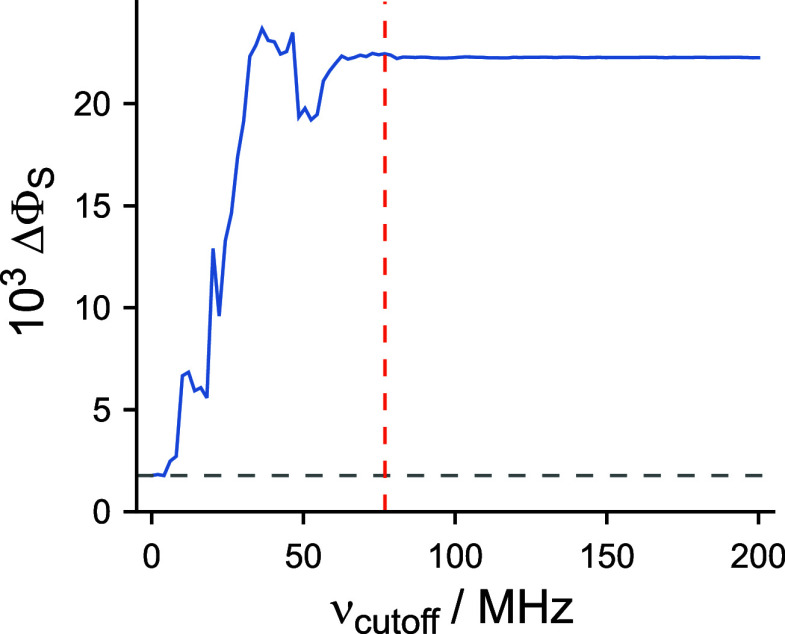
Changes in ΔΦ_S_ for a toy radical pair arising from modulations of hyperfine and dipolar interactions generated from a MD simulation of *Er*CRY4a (see text for details). The gray dashed line gives the value of ΔΦ_S_ calculated using time-independent interactions. The red dashed line shows the value of ν_max_, the difference between the largest and the smallest eigenvalues of the static part of the spin Hamiltonian (Equation 1).

Figure 8 shows the variation in ΔΦ_S_ as nuclear spins were added one at a time, roughly in order of decreasing strength of isotropic hyperfine interaction (Table S2). Data are shown for various combinations of static and dynamic hyperfine and dipolar interactions. As found in previous studies (e.g., refs 31, 42, and 57 ), ΔΦ_S_ falls dramatically as the number of nuclear spins is increased and when the dipolar interaction is included. Three additional trends can be discerned. First, the smallest values of ΔΦ_S_ are generally found when neither hyperfine nor dipolar interactions are time-dependent. In most cases, ΔΦ_S_ is increased when the dipolar interactions have a time-dependent component, and increased further when the hyperfine interactions are dynamic, whether the dipolar coupling is time-dependent or not. The greater sensitivity to modulation of the hyperfine interactions is probably because on average they are stronger than the dipolar couplings. The increase in the hypothetical compass sensitivity compared to the wholly static case confirms that the effects shown for the 2-nucleus spin system in Figure 7 are not limited to excessively simple systems.

**Figure 8:**
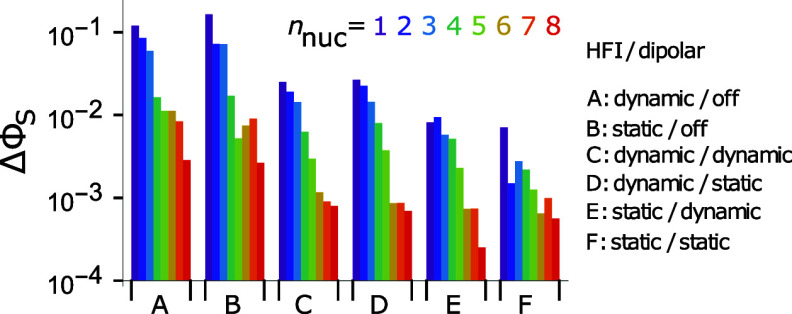
Dependence of the singlet yield anisotropy on the number of nuclear spins included in models of the FAD–TrpH radical pair. The hyperfine interactions were introduced one by one in approximate order of decreasing isotropic coupling: FN5, WNE1, FN10, WHE1, FH6, WHB1, FH8α, WHE3 (atom labels in Figure 1, F = FAD, W = TrpH). Hyperfine and dipolar interactions are static or dynamic, as indicated. In two cases, the dipolar interaction has been omitted (“off”). Note the logarithmic scale for ΔΦ_S_.

All the spin dynamics simulations reported above used Equation 3 to obtain Φ_S_(θ). For spin systems with more than 8 nuclei the SSE approach, Equation 7, is more efficient and allowed simulations of a model radical pair containing the 14 nuclei listed in Table S2. Figure 9 compares Φ_S_(θ) calculated for static and dynamic hyperfine and dipolar interactions. As before, inclusion of dynamic interactions increases ΔΦ_S_. In both cases the dependence on the magnetic field direction is very small showing that the downward trend in Figure 8 persists beyond the 8 nuclear spins that were possible to consider when using Equation 3.

Φ_S_(θ) is expected to be invariant to exact inversion of the magnetic-field direction, a property that follows from the time-reversal symmetry of spin operators.^58^ All previous simulations of anisotropic magnetic field effects using static spin Hamiltonians have confirmed this prediction (e.g., refs 9, 15, and 17 ). It was therefore unexpected to see (Figure 9, red) that Φ_S_(0) and Φ_S_(180°) are clearly different (nonoverlapping error bars) when the hyperfine and dipolar interactions are time-dependent. This seems to be a general property of time-dependent spin-Hamiltonians with anisotropic components. We have seen similar behavior in several types of spin dynamics simulations. 1) Using MD noise, lack of inversion symmetry was observed whether the calculation was performed using Equation 3 or Equation 7 for the time-dependence of the singlet fraction. It therefore does not result from the SU­(*Z*) trace sampling in the SSE approach. 2) It is not an artifact of the method used to extend the trajectories (illustrated in Figure 2e). 3) It occurs whether the hyperfine and dipolar interactions are modulated using MD noise or artificial broadband noise. 4) In the absence of noise of any kind, the inversion symmetry is also lost when the time-dependence of the spin Hamiltonian comes from an abrupt change in the strength of either the dipolar interaction, the hyperfine interaction, or the Zeeman interaction during the integration period *T* in Equation 4. 5) Asymmetry was found for both models of the FAD-TrpH radical pair, with 2 or 14 nuclear spins. Preliminary simulations suggest that the symmetry is restored if the reaction yields are averaged over a number of independent realizations of artificial noise. A selection of these calculations can be found in Section S6 of the Supporting Information. The conditions under which field-inversion asymmetry might be observed experimentally will be the subject of a future publication which will also discuss the possible relevance to avian magnetoreception where behavioral tests show that migratory songbirds have an inclination compass that is not affected by inversion of the geomagnetic field direction.^2,59^

**Figure 9:**
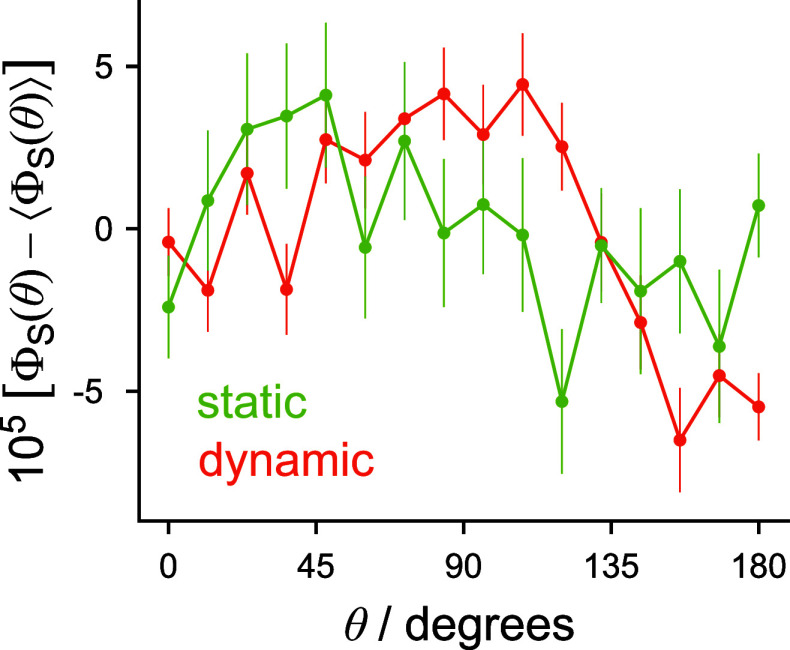
Singlet yield anisotropy, Φ_S_(θ) – ⟨Φ_S_(θ)⟩ as a function of magnetic field direction for a FAD–TrpH radical pair containing 14 nuclear spins, for which ν_max_ = 162.60 MHz. Calculations were performed using the SSE approach (Equation 6) with *M* = 96 randomly sampled SU­(*Z*) coherent states for each value of θ. The error bars represent the standard error of the mean of the singlet yields calculated using each of the 96 nuclear spin states. In the static case, the error bars for θ = 0 and θ = π overlap, consistent with the expected invariance of Φ_S_(θ) to inversion of the direction of the magnetic field.^58^

## 4 Discussion

We have simulated the direction-sensing performance of model radical pairs with explicitly time-dependent hyperfine and dipolar interactions. Three types of time-dependence have been considered: single-frequency amplitude modulation (Equation 8), broadband-noise modulation (Equation 9), and fluctuations calculated from a molecular dynamics simulation of European robin CRY4a. We shall refer to the last of these as “MD noise”. Spin dynamics simulations were performed for systems with up to 14 nuclear spins.

Several general conclusions can be drawn. First, the simulations confirm previous findings that inclusion of a dipolar interaction and increasing the number of nuclei coupled to the electrons both dramatically attenuate the reaction yield anisotropy, ΔΦ_S_.^27,31,57^ There have been several suggestions of mechanisms that could either circumvent these problems^57,60–64^ or provide a counteracting boost in ΔΦ_S_ ^30,32^ but so far none has been confirmed experimentally. Nevertheless, variants of the conventional radical pair mechanism with the potential to increase ΔΦ_S_ are of considerable interest.

Second, it is clear that quantitative conclusions on the viability of a cryptochrome-based magnetic sensor cannot be based on simplistic models in which magnetic interactions are omitted for computational convenience. Nor is it desirable to pretend that proteins at physiological temperatures are totally rigid and therefore that hyperfine and dipolar interactions are completely static. Spin relaxation and any other effects resulting from random thermal fluctuations in the atomic coordinates of a protein are not guaranteed to be negligible for radicals in a cellular setting.^29–31^

Third, previous approaches for the inclusion of motional effects in simulations of radical pair magnetoreceptors have either been phenomenological, for example using Lindblad operators^12,13,24^ or, more satisfactorily, have used Bloch–Redfield–Wangsness theory which treats the time-dependent magnetic interactions as perturbations of the static spin Hamiltonian.^30,31,40,42^ One limitation of the latter method in this context is that it is restricted to modeling the effects of specific motions, e.g. the libration of the flavin group around the N10–C1′ bond or fluctuations in the inter-radical separation. Another is that the matrix representation of the Redfield relaxation superoperator quickly becomes computationally prohibitive as the number of nuclear spins is increased. A third potential drawback is that the method may become unreliable if the fluctuating magnetic interactions do not satisfy the motional narrowing condition or cannot adequately be treated using second-order perturbation theory. The SSE approach, when combined with MD-noise, circumvents or ameliorates most of these issues. However, we stress that the results presented here were obtained using a single MD trajectory and so cannot be considered a comprehensive treatment of spin relaxation effects in cryptochrome.

Using a toy model of the FAD-TrpH pair, comprising just two anisotropic ^14^N hyperfine interactions, we have shown that as long as the modulation amplitude is not too large (in practice, less than 50%), modulation frequencies in excess of ν_max_ have little effect on the reaction yield Φ_S_(θ), where ν_max_ is the difference between the largest and smallest eigenvalues of the static component of the spin Hamiltonian (Equation 1). Evidently, fluctuations in local magnetic interactions need to be in resonance with transitions in the spin system to have a significant effect on the yield of the reaction products, presumably by causing coherences to dephase and/or by changing the populations of spin states.^65^ This finding implies that the higher frequency modulations of hyperfine and dipolar interactions (in practice >200 MHz) derived from MD trajectories can safely be removed allowing the state of the spin system to be propagated using a much longer time step (Equation 2).

We have also found that broadband-noise modulation of the internal magnetic interactions can increase ΔΦ_S_ and therefore the sensitivity to the direction of the geomagnetic field (Figures 3b,c and 5). Similar results were seen for MD noise (Figures 7–9) but not for single-frequency modulation (Figure 3a). For broadband noise, we calculated Φ_S_(θ) using the same set of noise parameters, ({Δ_*n*_, ν_*n*_, δ_*n*_}, *n* ∈[1, *N*] in Equation 9), for all directions of the geomagnetic field, θ, and then obtained ΔΦ_S_ as the difference between the maximum and minimum values of Φ_S_(θ) (Equation 6). To investigate the origin of this enhancement, the calculation of Φ_S_(θ) was repeated 16 times with a different set of noise parameters each time (Figure 5a). ΔΦ_S_ for each of the 16 traces was then calculated and the mean plotted against the amplitude of the broadband noise, σ_Δ_. The result is a boost in the apparent compass sensitivity, reaching a maximum enhancement of ∼ 300% when σ_Δ_≈ 0.3 (Figure 5d). Increases in ΔΦ_S_ when time-dependent magnetic interactions are included are also seen for the simulations shown in Figures 3b,c, 4, 7, and 9 showing that the effect is not specific to a particular form of noise or size of spin system.

At this point it is necessary to explain in a little more detail what exactly is modeled by Φ_S_(θ). As in earlier work, we assume that the magnetoreceptor molecules are located in photoreceptor cells distributed around the retina at the back of the eye.^36–38^ The cells are assumed to be identical, pencil-shaped and pointing at the pupil. Receptor molecules at different positions in the curved retina therefore experience different magnetic field directions by virtue of the orientations of the cells that contain them. The presumption is that comparison of the signals from receptor cells at different positions in the retina would allow the bird to determine the direction of the magnetic field. Simple geometry allows one to determine the field direction, θ, experienced by a radical pair at any point in the retina; spin dynamics simulations then give Φ_S_(θ).

This is a reasonable description when all the magnetic interactions are static, but care is needed when explicit time-dependence is included. In particular, using the same broadband or MD noise for different values of θ assumes that molecules in different cells in different parts of the retina have identical atomic motions. An arguably more realistic approach is to average the 16 calculations of Φ_S_(θ) (Figure 5a) and then determine the value of ΔΦ_S_ for this average signal. When this quantity is plotted against σ_Δ_ (Figure 5c), the directional sensitivity drops, ultimately to zero, as the noise amplitude is increased. This would be expected if the principal effect of the time-dependent local magnetic fields is to induce spin relaxation. Although the form of the signal shown in Figure 5c seems closer to reality, there may be circumstances in which the enhancement plotted in Figure 5d is relevant. As discussed by Smith et al.,^32^ who modeled sinusoidally driven radical motions, it is possible that radical pairs in different locations could undergo the same motions as a response to the abrupt charge separation following photon absorption.

Returning to Figure 5c, it appears that values of σ_Δ_ larger than 0.1 are required before the averaged ΔΦ_S_ drops significantly below its value for static interactions. To put this in context, Figure 6 shows that the standard deviation of the time-dependent components of the 14 strongest hyperfine interactions in *Er*CRY4a is, on average, ∼10% of the corresponding static components. Remembering the definition of σ_Δ_ as the standard deviation of the broadband-noise modulation of the hyperfine and dipolar interactions (Equation 9), Figure 5c therefore suggests that the harmful effects of random molecular motions in *Er*CRY4a may not be as serious a problem as one might have expected. It is difficult to know how much to read into this finding. It merits a deeper investigation, using MD simulations instead of artificial noise.

Finally on the theme of spin relaxation, it is clear from Figure 2c and Section S5 of the Supporting Information that there are components of the MD-noise that modulate hyperfine interactions at frequencies below 10 MHz. A detailed characterization of the noise would be required to determine how big an effect such motions could have on the anisotropy of the reaction yields. A longer simulation (5 μs or preferably 7 μs) with several replicas would be needed to get an impression of how reliably the most important low-frequency hyperfine and dipolar modulations had been captured. For example, the step changes in *A*_*zz*_(*t*) in Figure 2c at ∼150 and 300 ns are unlikely to be exactly reproduced in an independent simulation. This analysis will be the subject of a future study.

## 5 Conclusion

Our analysis of the sensitivity of radical pair magnetoreceptors to molecular motions in a protein is the most detailed to date. It combines explicitly time-dependent internal magnetic interactions obtained from MD simulations and electronic structure calculations with efficiently and accurately modeled spin dynamics of multinuclear electron–nuclear spin systems avoiding the limitations of perturbative spin relaxation theories. It has also identified the range of frequencies of molecular motions that are expected to have the greatest effects on the sensitivity to the direction of an Earth-strength magnetic field. We believe this work will pave the way for future studies that could provide significant additional insight into how Nature has evolved such a remarkable magnetic compass sense.

## Supplementary Material



## Notes

Hyperfine and dipolar tensors. Properties of ν_max_. *MolSpin* input files. Low-pass filtering of time series. Magnetic-field inversion simulations (PDF)
