# Supplementary material for: Magnetosensitivity of Model Flavin–Tryptophan Radical Pairs in a Dynamic Protein Environment
Source: J Phys Chem B. 2025 Jun 4;129(24):5937–47. doi: 10.1021/acs.jpcb.5c01187 (PMC12183739; doi:10.1021/acs.jpcb.5c01187)
Supplement: Supplementary file 1 [file jp5c01187_si_001.pdf]

Magnetosensitivity of model flavin-tryptophan radical pairs in a dynamic protein environment

Philip Benjamin, Luca Gerhards, Ilia A. Solov'yov, and P. J. Hore*

*Author for correspondence: peter.hore@chem.ox.ac.uk

Supplementary Information

Contents

S1. Hyperfine and dipolar tensors 1
S2. Maximum resonance frequency, ν_{max} 4
S3. Examples of *MolSpin* input files 5
S4. Low-pass filtering of time-series 7
S5. Correlation functions 8
S6. Magnetic-field inversion asymmetry 9
S6.1 Modulation of the hyperfine interaction using an Ornstein-Uhlenbeck process 9
S6.2 Sudden change in the strength of the Zeeman interaction 9
S6.3 Sudden change in the hyperfine interaction 10
S6.4 Sudden change in the strength of the dipolar interaction 10
References 11

S1. Hyperfine and dipolar tensors

Table S1. Static components of the magnetic interactions (in mT) in the toy model used for Figures 3-5. See Figure 1 for atom labels and representations of the hyperfine tensors. These data come from Refs [1-3].

**$FAD^{\bullet-}$ hyperfine interaction: N5**	$\begin{pmatrix} -0.0989 & 0 & 0 \\ 0 & -0.0989 & 0 \\ 0 & 0 & 1.7569 \end{pmatrix}$
**$TrpH^{\bullet+}$ hyperfine interaction: N1**	$\begin{pmatrix} -0.0336 & 0.0924 & -0.1354 \\ 0.0924 & 0.3303 & -0.5318 \\ -0.13540 & -0.5318 & 0.6680 \end{pmatrix}$
**Dipolar interaction**	$\begin{pmatrix} 0.0390 & -0.4329 & 0.1752 \\ -0.4329 & -0.2786 & 0.2509 \\ 0.1752 & 0.2509 & 0.2397 \end{pmatrix}$

Table S2. Static components of the magnetic interactions (in mT) used for Figures 6-8. See Figure 1 for atom labels and representations of the hyperfine tensors. These data are the time-averages calculated from the 953-ns MD simulation of *Er*Cry4a.

**$FAD^{\bullet -}$ hyperfine interactions**	
N5	$\begin{pmatrix} -0.01470 & 0 & 0 \\ 0 & 0.00280 & 0 \\ 0 & 0 & 1.71925 \end{pmatrix}$
N10	$\begin{pmatrix} 0.00501 & 0.00019 & -0.00812 \\ 0.00019 & 0.0155 & 0.00777 \\ -0.00812 & 0.00777 & 0.57609 \end{pmatrix}$
H6	$\begin{pmatrix} -0.40695 & 0.10278 & -0.00382 \\ 0.10278 & -0.2190 & -0.01809 \\ -0.00382 & -0.01809 & -0.40227 \end{pmatrix}$
H81	$\begin{pmatrix} 0.4395 & -0.03832 & 0.00138 \\ -0.03832 & 0.43469 & -0.0011 \\ 0.00138 & -0.0011 & 0.39761 \end{pmatrix}$
H82	$\begin{pmatrix} 0.43386 & -0.03828 & 0.00146 \\ -0.03828 & 0.42935 & -0.00119 \\ 0.00146 & -0.00119 & 0.39211 \end{pmatrix}$
H83	$\begin{pmatrix} 0.43428 & -0.03821 & 0.00128 \\ -0.03821 & 0.43026 & -0.00116 \\ 0.00128 & -0.00116 & 0.39273 \end{pmatrix}$
H1′	$\begin{pmatrix} 0.11098 & 0.00401 & 0.00248 \\ 0.00401 & 0.23254 & 0.02574 \\ 0.00248 & 0.02574 & 0.10228 \end{pmatrix}$

**TrpH$^{\bullet+}$ hyperfine interactions**	
NE1	$\begin{pmatrix} 0.54818 & -0.03906 & -0.45739 \\ -0.03906 & 0.04261 & 0.04645 \\ -0.45739 & 0.04645 & 0.45515 \end{pmatrix}$
HE1	$\begin{pmatrix} -0.26593 & 0.15885 & 0.29687 \\ 0.15885 & -0.74793 & 0.21922 \\ 0.29687 & 0.21922 & -0.19632 \end{pmatrix}$
HE3	$\begin{pmatrix} -0.45986 & 0.1964 & 0.12575 \\ 0.1964 & -0.70434 & 0.18166 \\ 0.12575 & 0.18166 & -0.54132 \end{pmatrix}$
HZ2	$\begin{pmatrix} -0.26731 & 0.12995 & 0.11326 \\ 0.12995 & -0.43698 & 0.12014 \\ 0.11326 & 0.12014 & -0.33918 \end{pmatrix}$
HB1	$\begin{pmatrix} 1.22544 & 0.02909 & 0.07086 \\ 0.02909 & 1.28116 & 0.04853 \\ 0.07086 & 0.04853 & 1.33955 \end{pmatrix}$
HD1	$\begin{pmatrix} -0.44082 & -0.19500 & 0.06364 \\ -0.19500 & -0.32402 & -0.22874 \\ 0.06364 & -0.22874 & -0.37996 \end{pmatrix}$
HH2	$\begin{pmatrix} -0.38371 & -0.05145 & -0.04833 \\ -0.05145 & -0.06123 & -0.06946 \\ -0.04833 & -0.06946 & -0.35530 \end{pmatrix}$

**Dipolar interaction**	$\begin{pmatrix} -0.32445 & -0.1989 & 0.42318 \\ -0.1989 & 0.26868 & 0.12887 \\ 0.42318 & 0.12887 & 0.05578 \end{pmatrix}$

S2. Maximum resonance frequency, ν_{max}

ν_{max} (Figures 3, 4, 7, and 9) is the frequency corresponding to the difference between the highest and lowest eigenvalues of the static spin Hamiltonian of the radical pair. For both of the spin systems considered here, ν_{max} is dominated by the hyperfine interactions as can be seen from Tables S3 and S4 which show the effects of changing the strength of the dipolar interaction, keeping the hyperfine interactions fixed. The dependence on D is fairly minor for both spin systems.

The same is true of the Zeeman interaction. For the 2-nucleus model, ν_{max} is 81.5 MHz in the absence of a static magnetic field, and 81.7-83.9 MHz when the field, B, is 50 μT, depending on its direction.

Table S3. Dependence of ν_{max} on the radical-radical separation (r) and dipolar coupling (D) for the 2-nucleus model. $B = 0$.

r / nm	D / mT	ν_{max} / MHz
1.5	−0.824	84.0
1.6	−0.679	82.7
1.7	−0.566	81.8
1.8	−0.477	81.3
1.9	−0.405	80.9
2.0	−0.347	80.6

Table S4. Dependence of ν_{max} on the radical-radical separation (r) and dipolar coupling (D) for the 14- nucleus model. $B = 0$.

r / nm	D / mT	ν_{max} / MHz
1.5	−0.824	164.8
1.6	−0.679	163.2
1.7	−0.566	162.1
1.8	−0.477	161.3
1.9	−0.405	160.7
2.0	−0.347	160.2

Further information on the significance of ν_{max} in the context of the magnetic disorientation of migratory birds in weak radiofrequency magnetic fields can be found in Refs [4-6].

S3. Examples of *MolSpin* input files

StaticInput.msd: input file used to calculate $\Delta\Phi_S$ for radical pairs with static magnetic interactions (Figures 3-5).

StaticInput.msd

```
SpinSystem RPSystem{
    //Electron spins
    Spin RPElectron1{type = electron; tensor = isotropic(2.0023); spin = 1/2;}
    Spin RPElectron2{type = electron; tensor = isotropic(2.0023); spin = 1/2;}
    //FAD nuclear spin
    Spin N5{type = nucleus; spin = 1;tensor = isotropic(1);}
    //TRP nuclear spin
    Spin N1{type = nucleus; spin = 1;tensor = isotropic(1);}
    //Dipolar Interaction
    Interaction Dipolar{type=doublespin; ignoretensors=true; prefactor = 0.0020023;
                        group1=RPElectron1; group2=RPElectron2;
                        tensor=matrix("0.03893 -0.43289 0.17518;
                                      -0.43289 -0.27864 0.25086;
                                       0.17518 0.25086 0.23971");}
    //Zeeman Interaction
    Interaction Zeeman_Static{type=zeeman; prefactor=0.001;
                                spins=RPElectron1, RPElectron2; field="0.0 0.0 0.05";}
    //Hyperfine Interaction
    Interaction HFI_N5{type=hyperfine; group1=RPElectron1;
                        group2=N5; prefactor=0.001;
                        tensor=matrix("-0.09890 0.00000 0.00000;
                                        0.00000 -0.09890 0.00000;
                                        0.00000 0.00000 1.75690");}
    Interaction HFI_N1{type=hyperfine; group1=RPElectron2; group2=N1; prefactor=0.001;
                        tensor=matrix("-0.03360 0.09240 -0.13540;
                                        0.09240 0.33030 -0.53180;
                                       -0.13540 -0.53180 0.66800");}
    //Define Spin States
    State Singlet{spins(RPElectron1, RPElectron2) = |1/2,-1/2> - |-1/2,1/2>;}
    State T0        // |T0>
    {
            spins(RPElectron1,RPElectron2) = |1/2,-1/2> + |-1/2,1/2>;
    }
    State Tp        // |T+>
    {
            spin(RPElectron2) = |1/2>;
            spin(RPElectron1) = |1/2>;
    }
    State Tm        // |T->
    {
            spin(RPElectron2) = |-1/2>;
            spin(RPElectron1) = |-1/2>;
    }

    //Define recombination rate
    Transition Product1{type = sink;source = Singlet;rate = 0.001;}
    Transition Product2{type = sink;source = T0;rate = 0.001;}
    Transition Product3{type = sink;source = Tp;rate = 0.001;}
    Transition Product4{type = sink;source = Tm;rate = 0.001;}
}
//Rotate the field
Settings{
        Settings general {steps = 31; notifications = details;}
        Action scan{
                type = rotatevector;
                vector = RPSystem.Zeeman_static.field;
                axis = "0 1 0";
                value = 6;
        }
}
//Calculate quantum yields for each orientation of the field
Run{
    Task main{
            type = "statichs-direct-yields"; logfile = "log_static.log"; datafile = "dat_static.dat";
            transitionyields = true; initialstate = singlet; totaltime = 5000; timestep = 0.5;
            propagationmethod = "autoexpm"; precision = "single"; yieldcorrections = true;
            }
}
```

DynamicInput.msd: input files similar to this were used to calculate $\Delta\Phi_S$ for radical pairs with dynamic magnetic interactions. The time-dependent data were input to *MolSpin* as *.mst* trajectory files.

DynamicInput.msd

```
SpinSystem RPSystem{
    //Electron spins
    Spin RPElectron1{type = electron; tensor = isotropic(2.0023); spin = 1/2;}
    Spin RPElectron2{type = electron; tensor = isotropic(2.0023); spin = 1/2;}
    //FAD nuclear spin
    Spin N5{type = nucleus; spin = 1; tensor = isotropic(1);}
    //TRP nuclear spin
    Spin N1{type = nucleus; spin = 1; tensor = isotropic(1);}
    //Dipolar Interaction
    Interaction Dipolar{type=doublespin; group1=RPElectron1; group2=RPElectron2; prefactor=0.0020023;
                        tensor=trajectory("Dipolar.mst"); trajectory="Dipolar.mst";}
    //Zeeman Interaction
    Interaction Zeeman_Static{type=zeeman; prefactor=0.001; spins=RPElectron1, RPElectron2;
                        field="0.0 0.0 0.05";}
    //Hyperfine Interaction
    Interaction HFI_N5{type=hyperfine; group1=RPElectron1; group2=N5; prefactor=0.001;
                        tensor=trajectory("N5.mst"); trajectory="N5.mst";}
    Interaction HFI_N1{type=hyperfine; group1=RPElectron2; group2=N1; prefactor=0.001;
                        tensor=trajectory("N1.mst"); trajectory="N1.mst";}
    //Define Spin States
    State Singlet{spins(RPElectron1,RPElectron2) = |1/2,-1/2> - |-1/2,1/2>;}
    State T0        // |T0>
    {
            spins(RPElectron1,RPElectron2) = |1/2,-1/2> + |-1/2,1/2>;
    }
    State Tp        // |T+>
    {
            spin(RPElectron2) = |1/2>;
            spin(RPElectron1) = |1/2>;
    }
    State Tm        // |T->
    {
            spin(RPElectron2) = |-1/2>;
            spin(RPElectron1) = |-1/2>;
    }
    //Define recombination rate
    Transition Product1{type = sink;source = Singlet;rate = 0.001;}
    Transition Product2{type = sink;source = T0;rate = 0.001;}
    Transition Product3{type = sink;source = Tp;rate = 0.001;}
    Transition Product4{type = sink;source = Tm;rate = 0.001;}
}
//Rotate the field
Settings{
        Settings general {steps = 31; notifications = details;}
        Action scan{
                type = rotatevector;
                vector = RPSystem.Zeeman_static.field;
                axis = "0 1 0";
                value = 6;
        }
}
//Calculate quantum yields for each orientation of the field
Run{
    Task main{
            type = "dynamichs-direct-yields"; logfile = "log_static.log"; datafile =
            "dat_static.dat"; transitionyields = true; initialstate = singlet; totaltime = 5000;
            timestep = 0.5; propagationmethod = "autoexpm"; precision = "single"; yieldcorrections = true;
            }
}
```

More information on compiling *MolSpin* and running the input files can be found at https://www.molspin.eu/manual.

Pre-release versions at https://github.com/Das0Mann/MolSpin contain methods for generating artificial time-dependent hyperfine and dipolar interactions, as discussed in the main text, without the need for user-generated trajectory files.

S4. Low-pass filtering of time-series

The Fourier transforms used to remove high-frequency components from the time-dependence of the hyperfine and dipolar interactions (illustrated in Figure 2 in the main text) were performed without apodization or zero-padding. As a check that this procedure does not significantly distort the remaining low-frequency components, Figure S1 compares data obtained in this way with a low-pass filtered version obtained using a Blackman window with filter kernel length = 1000 and cut-off frequency = 200 MHz (LowpassFilter function in *Mathematica*). The difference between the two is minimal.

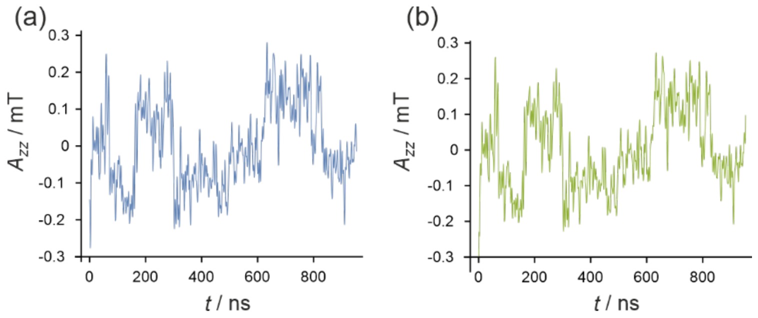

Figure S1. (**a**) Time-dependence of $A_{zz}(\mathrm{N5})$ produced by removing components at frequencies above 200 MHz using the procedure described in the main text. This plot is identical to Figure 2(b) apart from the time range ($0 \le t \le 953$ ns instead of $0 \le t \le 500$ ns). (**b**) The same time-series obtained using a low-pass filter with a Blackman window. In both cases, the mean signal amplitude was subtracted prior to processing.

Figure S2 compares the Fourier transforms of the time-series in Figure 2(c) calculated (a) with no apodization or zero-padding) and (b) with apodization (Blackman filter) and zero-padding to double the time-series.

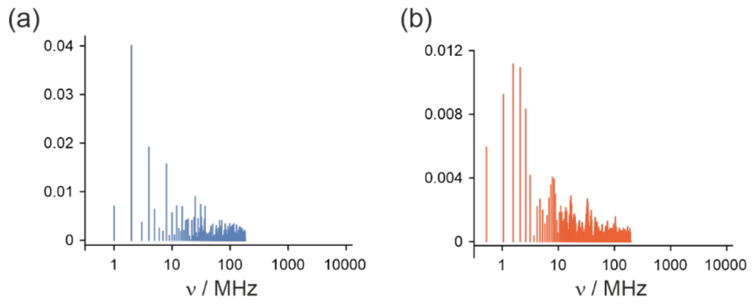

Figure S2. Power spectra for $A_{zz}(\mathrm{N5})$ obtained (**a**) without and (**b**) with apodization and zero-padding.

S5. Correlation functions

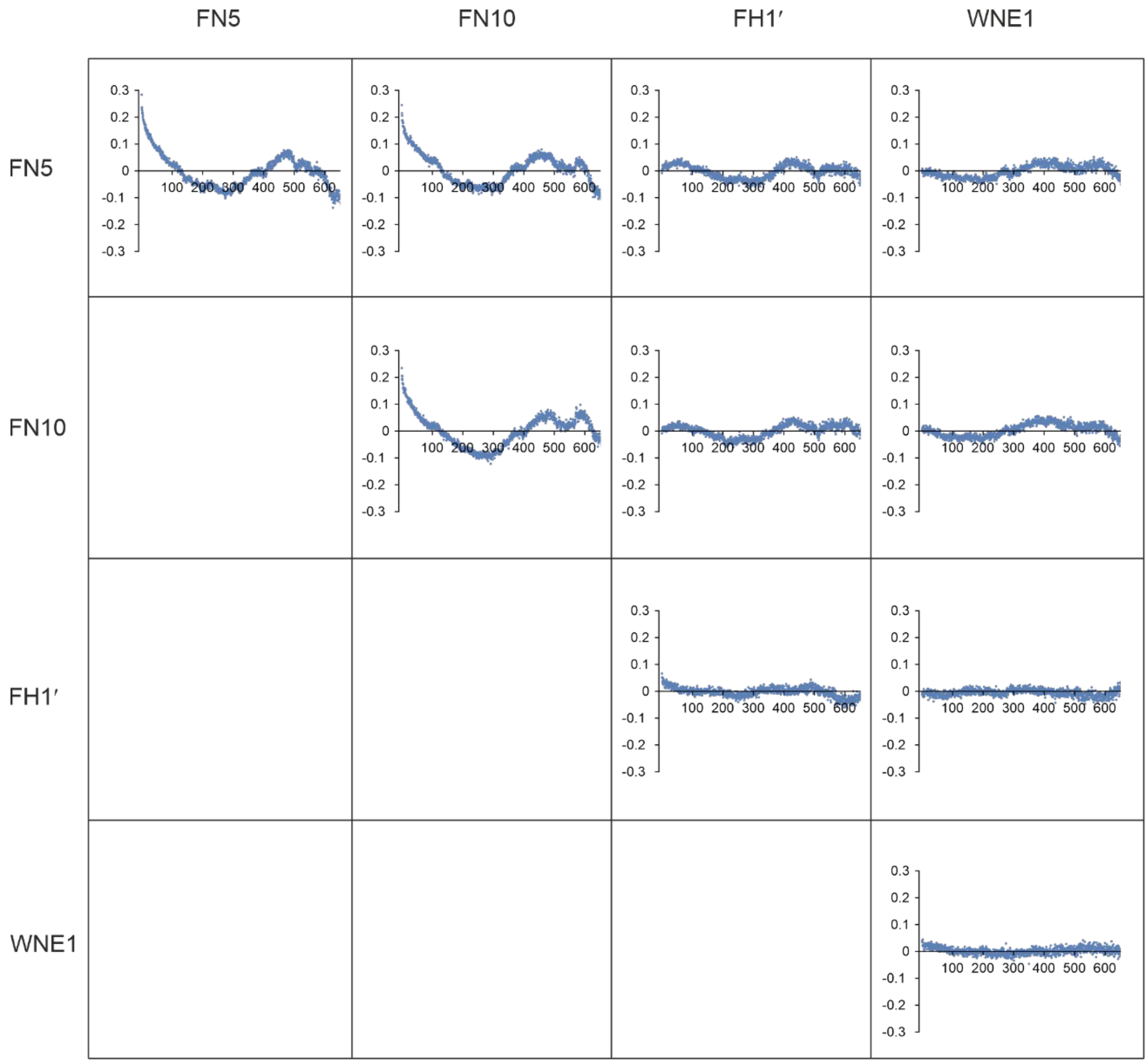

Figure S3. Auto- and cross-correlation functions for the *zz* components of the hyperfine tensors of N5, N10, and H1′ in FAD$^{\bullet-}$ and NE1 in TrpH$^{\bullet+}$. The horizontal axes are in nanoseconds. Calculated from raw data (δt = 50 ps, N = 19,068) using

$$cf(m, \mathbf{x}, \mathbf{y}) = \frac{1}{N-m} \sum_{i=1}^{N-m} x_i y_{m+i}$$

with **x** and **y** shifted and rescaled to have zero mean and unit variance.

S6. Magnetic-field inversion asymmetry

Below, we give the results of a few simulations showing that a time-dependent spin Hamiltonian can destroy the invariance of the singlet yield, Φ_S, to inversion of the external magnetic field. In all cases, the spin system is a radical pair with a single spin-½ nucleus in one of the radicals. θ (co-latitude) and ϕ (azimuth) specify the magnetic field axis in the same coordinate system as the hyperfine tensor. k is the recombination rate constant. D is the dipolar interaction parameter. B is the strength of the magnetic field.

S6.1 Modulation of the hyperfine interaction using an Ornstein-Uhlenbeck process

$\Phi_S(B)$ and $\Phi_S(-B)$ were calculated using Equations (2)-(4) with δt = 10 ps and T = 10 μs. Exponentially correlated noise from a Gaussian random process (Ornstein-Uhlenbeck) with zero mean, standard deviation σ = 0.04 mT, and correlation time τ_C was added to each of the elements of the hyperfine tensor:

$$A_{ij}(t) = A_{ij} + a_{ij}(t) \text{ with } A_{ij}(t) = A_{ji}(t), \tag{1}$$

$$\langle a_{ij}(t)a_{ij}(t+\tau)\rangle = \sigma^2 \exp(-\tau/\tau_C). \tag{2}$$

The same set of noise values was used to calculate $\Phi_S(B)$ and $\Phi_S(-B)$.

Parameters: $B = 1$ mT; $D = 0$; $k = 1.0 \times 10^6$ s^{-1}; $\theta = 0.3420$ rad; $\phi = 2.9395$ rad;

$$\mathbf{A} = \begin{pmatrix} 0.2147 & -0.1976 & 0.1958 \\ -0.1976 & -0.0936 & 0.0538 \\ 0.1958 & 0.0538 & -0.0999 \end{pmatrix} \text{ mT.}$$

Results:

τ_C / s	10^{-10}	10^{-9}	10^{-8}	10^{-7}	10^{-6}	10^{-5}
$\Phi_S(B)-\Phi_S(-B)$	-1.48×10^{-3}	-1.09×10^{-2}	-4.42×10^{-3}	6.69×10^{-3}	1.80×10^{-3}	3.66×10^{-4}

Without added noise $\Phi_S(B) - \Phi_S(-B) = 3.31\times10^{-11} \approx 0$.

S6.2 Sudden change in the strength of the Zeeman interaction

Here, the time-dependence of the spin Hamiltonian came from an abrupt change in the strength of the external magnetic field, from B_1 to B_2, at t = 1 μs. No added noise.

Parameters: $D = -0.5$ mT; $k = 1.0 \times 10^6$ s^{-1}; $\theta = 0.3420$ rad; $\phi = 2.9395$ rad;

$$\mathbf{A} = \begin{pmatrix} 0.2147 & -0.1976 & 0.1958 \\ -0.1976 & -0.0936 & 0.0538 \\ 0.1958 & 0.0538 & -0.0999 \end{pmatrix} \text{ mT.}$$

Results:

B_1 / mT	0.05	10.0	0.05	10.0
B_2 / mT	0.05	10.0	10.0	0.05
$\Phi_S(B_1,B_2)-\Phi_S(-B_1,-B_2)$	$< 10^{-14}$	$< 10^{-14}$	-8.55×10^{-5}	-1.49×10^{-3}

The singlet yield is only invariant to inversion of the magnetic field when $B_1 = B_2$.

S6.3 Sudden change in the hyperfine interaction

Here, the time-dependence of the spin Hamiltonian came from an abrupt change in the hyperfine tensor, from $\mathbf{A}_1$ to $\mathbf{A}_2$ at $t = 1$ μs. No added noise.

Parameters: $B = 0.05$ mT; $D = -0.5$ mT; $k = 1.0 \times 10^6$ s^{-1}; $\theta = 0.3420$ rad; $\phi = 2.9395$ rad;

$$\boldsymbol{\alpha}_1 = \begin{pmatrix} 0.2147 & -0.1976 & 0.1958 \\ -0.1976 & -0.0936 & 0.0538 \\ 0.1958 & 0.0538 & -0.0999 \end{pmatrix} \text{mT}; \quad \boldsymbol{\alpha}_2 = \begin{pmatrix} -0.02284 & 0.00537 & 0.00026 \\ 0.00537 & -0.01448 & -0.01894 \\ 0.00026 & -0.01894 & 0.00972 \end{pmatrix} \text{mT}.$$

Results:

$\mathbf{A}_1$	$\boldsymbol{\alpha}_1$	$\boldsymbol{\alpha}_2$	$\boldsymbol{\alpha}_1$	$\boldsymbol{\alpha}_2$
$\mathbf{A}_2$	$\boldsymbol{\alpha}_1$	$\boldsymbol{\alpha}_2$	$\boldsymbol{\alpha}_2$	$\boldsymbol{\alpha}_1$
$\Phi_S(B)-\Phi_S(-B)$	$< 10^{-14}$	$< 10^{-14}$	3.71×10^{-3}	-4.57×10^{-3}

The singlet yield is only invariant to inversion of the magnetic field when $\mathbf{A}_1 = \mathbf{A}_2$.

S6.4 Sudden change in the strength of the dipolar interaction

Here, the time-dependence of the spin Hamiltonian came from an abrupt change in the strength of the dipolar interaction, from D_1 to D_2 at $t = 1$ μs. No added noise.

Parameters: $B = 1.0$ mT; $k = 1.0 \times 10^6$ s^{-1}; $\theta = 0.3420$ rad; $\phi = 2.9395$ rad;

$$\mathbf{A} = \begin{pmatrix} 0.2147 & -0.1976 & 0.1958 \\ -0.1976 & -0.0936 & 0.0538 \\ 0.1958 & 0.0538 & -0.0999 \end{pmatrix} \text{mT}.$$

Results:

D_1 / mT	−1.0	−2.0	−1.0	−2.0
D_2 / mT	−1.0	−2.0	−2.0	−1.0
$\Phi_S(B)-\Phi_S(-B)$	$< 10^{-14}$	$< 10^{-14}$	-6.97×10^{-3}	-3.84×10^{-3}

The singlet yield is only invariant to inversion of the magnetic field when $D_1 = D_2$.

References

1. Lee, A.A., Lau, J.C.S., Hogben, H.J., Biskup, T., Kattnig, D.R., and Hore, P.J. (2014). Alternative radical pairs for cryptochrome-based magnetoreception. J. R. Soc. Interface *11*, 20131063.
2. Hiscock, H.G., Kattnig, D.R., Manolopoulos, D.E., and Hore, P.J. (2016). Floquet theory of radical pairs in radiofrequency magnetic fields. J. Chem. Phys. *145*, 124117.
3. Wong, S.Y., Benjamin, P., and Hore, P.J. (2023). Magnetic field effects on radical pair reactions: estimation of $B_{1/2}$ for flavin-tryptophan radical pairs in cryptochromes. Phys. Chem. Chem. Phys. *25*, 975-982.
4. Hiscock, H.G., Mouritsen, H., Manolopoulos, D.E., and Hore, P.J. (2017). Disruption of magnetic compass orientation in migratory birds by radiofrequency electromagnetic fields. Biophys. J. *113*, 1475-1484.
5. Leberecht, B., Kobylkov, D., Karwinkel, T., Doge, S., Burnus, L., Wong, S.Y., Apte, S., Haase, K., Musielak, I., Chetverikova, R., et al. (2022). Broadband 75-85 MHz radiofrequency fields disrupt magnetic compass orientation in night-migratory songbirds consistent with a flavin-based radical pair magnetoreceptor. J. Comp. Physiol. A *208*, 97-106.
6. Leberecht, B., Wong, S.Y., Satish, B., Döge, S., Hindman, J., Venkatraman, L., Apte, S., Haase, K., Musielak, I., Dautaj, G., et al. (2023). Upper bound for broadband radiofrequency field disruption of magnetic compass orientation in night-migratory songbirds. Proc. Natl. Acad. Sci. USA *120*, 2301153120.
